# Norovirus transmission dynamics: a modelling review

**DOI:** 10.1017/S0950268817002692

**Published:** 2017-12-22

**Authors:** K. A. M. GAYTHORPE, C. L. TROTTER, B. LOPMAN, M. STEELE, A. J. K. CONLAN

**Affiliations:** 1 Department of Veterinary Medicine, University of Cambridge, Cambridge, UK; 2 Department of Epidemiology, Emory University, Atlanta, Georgia, USA

**Keywords:** Basic reproduction number, estimating disease prevalence, mathematical modelling, norovirus, transmission

## Abstract

Norovirus is one of the leading causes of viral gastroenteritis worldwide and responsible for substantial morbidity, mortality and healthcare costs. To further understanding of the epidemiology and control of norovirus, there has been much recent interest in describing the transmission dynamics of norovirus through mathematical models. In this study, we review the current modelling approaches for norovirus transmission. We examine the data and methods used to estimate these models that vary structurally and parametrically between different epidemiological contexts. Many of the existing studies at population level have focused on the same case notification dataset, whereas models from outbreak settings are highly specific and difficult to generalise. In this review, we explore the consistency in the description of norovirus transmission dynamics and the robustness of parameter estimates between studies. In particular, we find that there is considerable variability in estimates of key parameters such as the basic reproduction number, which may mean that the effort required to control norovirus at the population level may currently be underestimated.

## INTRODUCTION

Norovirus is an important cause of acute gastroenteritis (AGE) worldwide, and is associated with considerable morbidity, mortality and healthcare costs. In Africa and South East Asia, almost a quarter of deaths in children under 5 are caused by AGE [1, 2]. The majority of non-bacterial outbreaks, and an estimated 18% of all endemic AGE, are caused by norovirus [1, 3–5]. The virus was first described in 1969 and first identified in 1972 [6, 7]. Today, norovirus is estimated to cost healthcare services and patients 81 million a year in the UK alone [8, 9].

Noroviruses are a genus within the *Caliciviridae* family which is divided into five genogroups (GI–GV) [10–12]. GII is the most prevalent genogroup worldwide, accounting for over 81% of all outbreaks in the USA reported to Calicinet in 2017 [13]. Genotype II, type 4 viruses are the predominant strain and the leading cause of epidemic AGE in children and adults worldwide. It is responsible for 70–80% of reported outbreaks in the last decade [4, 11, 12, 14]. There may be considerable antigenic variation in GII.4 with a new pandemic strain appearing every 2–3 years [15, 16]. However, the GII.17 genotype has recently become the predominant strain in some parts of Asia [17], and 2016 saw the emergence of new recombinant GII P16-GII.2 in Germany [18].

The main transmission route for norovirus is faecal–oral, particularly for the epidemic strain GII.4, and transmission may be enhanced by vomiting incidents [3, 19–21]. However, other strains are more commonly associated with environmentally transmitted outbreaks [4, 22]. This can be through contamination of food, water or surfaces and there have been examples where outbreaks have been driven by environmental transmission both on cruise ships and air planes [9, 23–25]. There is a dose–response relationship whereupon individuals exposed to a higher number of viral particles experience a higher attack rate [25]. However, the infectious dose is low, with a 49% probability of infection from one particle, which means the dose response is unlikely to be rate limiting [26, 27].

Norovirus transmission and resulting illness can be exacerbated by many factors. The virus exhibits strong seasonality with over half of cases occurring in winter months [28]. There are both environmental drivers and population behaviours cited as reasons for seasonal variation. For example, norovirus is more readily transmitted in colder temperatures and may be facilitated by increased rainfall [28–30]. Other population attributes can also affect the severity of norovirus outbreaks. The virus affects all age groups but the highest incidence is found in children under 5 years old [9, 31]. Serological surveys also suggest that the first norovirus infection occurs early in life [5, 32–35]. Elderly and immunocompromised individuals are more likely to experience severe complications and death [36, 37]. As such, whilst infection is self-limiting in healthy individuals, the effects experienced by certain high-risk groups can be severe. It has also been found that these groups can have far longer shedding durations which could prolong an outbreak [31, 38, 39].

A vital tool for understanding disease transmission and predicting the effectiveness of new control measures is mathematical modelling. Mathematical models must strike a balance between the biological realism of their representation of the natural history of infection, and the strength of their link to data [40–42]. Models of noroviruses developed at different scales, from the outbreak to population levels, have found a remarkable variability in the estimates of reproduction numbers. In this review, we examine the current status of mathematical modelling in norovirus transmission with particular focus on estimates of the reproduction number. We highlight knowledge gaps and suggest important new directions for development.

## AIMS

Published models of norovirus transmission demonstrate a broad range of relevant settings and characteristics, from local transmission within healthcare settings to the population level. The first aim of this paper is to review model structures and data in norovirus modelling and highlight areas that are relatively unexplored.

The second aim of this review is to compare and contrast the current estimates of the basic reproduction number for norovirus. Perhaps the most important epidemiological parameter, the basic reproduction number quantifies the risk of an epidemic, the potential for spread of a disease and the level of effort required for control.

The basic reproduction number, or *R*_0_, is defined as the *expected number of secondary infections per generation given one infected individual is introduced to an entirely susceptible population* [42].

Some authors, including those from studies considered in this review, do not clearly distinguish *R*_0_ from effective reproduction numbers. Effective reproduction numbers, *R*_E_, can be defined for partially susceptible populations where the potential for transmission of a pathogen is limited by ‘herd immunity’. When the population is well mixed, the relationship between effective and basic reproduction numbers is linear, where *R*_E_ = (*S*/*N*)*R*_0_ if *S* is the number of susceptible individuals in a population of size *N*. We would expect variation in the values of *R*_0_ and *R*_E_ for different settings. A key objective of this review is to clarify this distinction between and separate the likely range of these key measures for norovirus.

## REVIEW METHODS

A literature search was conducted on articles published before April 2017 using the search engines Google scholar and PubMed with additional results found through reference tracing, see Supplementary Material for flow diagram referring to PubMed search and search terms. We did not aim to conduct a systematic review, but did refer to PRISMA guidelines to follow good practice. The key words used in the search included ‘Norovirus’, ‘Norwalk’, ‘models, theoretical’, ‘transmission dynamics’, ‘R0’ and ‘basic reproduction number’ with a full list of MeSH terms as specified in the Supplementary Material. Only English language papers were included in the literature review, with a focus on mathematical modelling studies of norovirus transmission and estimation of key quantities such as reproduction numbers. After our initial review was conducted, the key reference list was compared to a separate review commissioned by Takeda Pharmaceuticals and performed by Amaris (10 August 2015) to check for additional references. The Amaris review searched MEDLINE and Cohrane databases, with the aim of identifying clinical and epidemiological parameters used in economic and epidemiological models for norovirus, and yielded 13 epidemiological modelling and six economic modelling studies. From this, we identified one additional paper [43], for inclusion in our review.

**Fig. 1.**
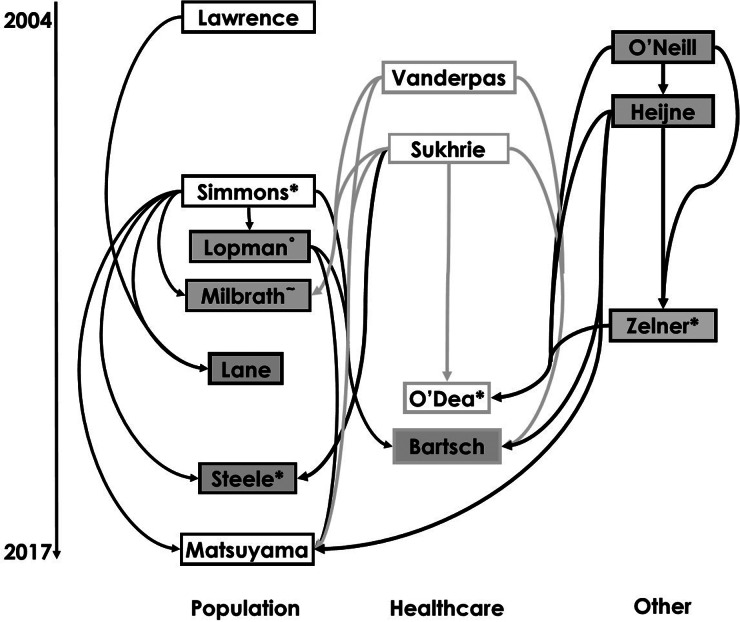
Diagram of citations between reviewed works divided by setting [10, 20, 21, 31, 35, 43, 46–48, 52–54, 59, 69]. Boxes show first authors of each study. Arrows denote that the study may have been influenced by earlier studies, established through citation, and arrow colour varies for ease of reading. Boxes with white backgrounds indicate a study has estimated and provided a novel value of a reproduction number. Superscript symbols denote co-authorship with * indicating co-authorship with Lopman; ^°^ Simmons and ^~^ Zelner.

The reviewed works included in our study are shown in a citation map in Figure 1 with search strategy summarised in Figure S1 in the Supplementary Material. This visualisation allows us to trace how model structures and assumptions have flowed through different norovirus modelling studies.

## GENERAL FEATURES

**Fig. 2.**
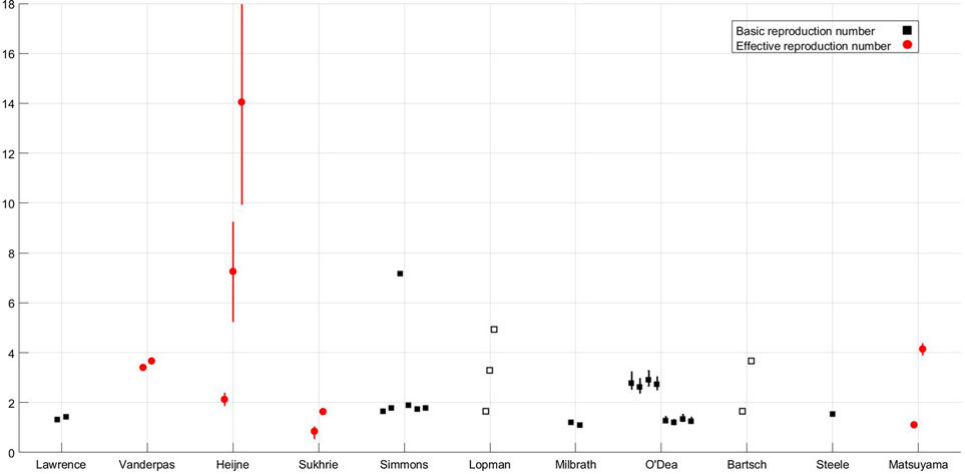
Reproduction number values for the subset of studies, shown in Fig. 1, where reproduction numbers are *explicitly* mentioned; the *x*-axis denotes first author [10, 21, 31, 35, 43, 47, 48, 52–54, 69]. Squares denote individual values of the basic reproduction number according to the definition given in the Aims section; circles denote individual values of the effective reproduction number according to the definition given in the Aims section. Filled shapes denote that the value was estimated, empty shapes denote that the value was assumed and lines denote 95% confidence interval ranges if provided. Where mutliple reproduction number values are estimated for different situations, detailed in text, all values are shown.

The majority of norovirus transmission models take a compartmental approach where the population is split by disease state. Most commonly, the population is divided into susceptible, infected and recovered classes (SIR). A latent, or exposed, compartment (E) is also commonly included in models for norovirus. The latent compartment is necessary to examine the potential effectiveness of controls targeting infected individuals [44]. Within such an SEIR model, the population is distributed into four states where susceptible individuals may become exposed if they come into contact with infected individuals. Once exposed, individuals progress to be infectious and then recovered or removed. In the simplest case, transitions are assumed to occur at a rate inversely proportional to the duration of time spent in each compartment [45]. The number of individuals in each disease state at any time can be described by a set of coupled ordinary differential equations, see (1).
1
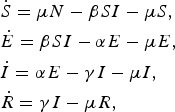

where *μ* is the per-capita birth or death rate; *β*, the transmission rate; *α*, the rate of latency loss and *γ*, the recovery rate. This basic framework can be adapted to include age or spatial structure, stochastic transitions or additional infectious states such as asymptomatic infection. The duration of immunity to re-infection with norovirus is likely to be short lived. This basic SEIR structure may suffice for describing single epidemics of norovirus, but modelling the longer term between season dynamics of norovirus requires additional flows to describe this waning of immunity. We include a table detailing model features, data used and form of any estimated reproduction number, see Table 1. The values of current reproduction number estimates are shown in Figure 2, with multiple values shown where a study has used different models to arrive at their estimates.

**Table 1.** Summary of reviewed studies and their reproduction number estimates where appropriate [10, 20, 31, 35, 43, 46, 48, 52–54, 59, 69]

Reference	Setting	Data	Model type(s)	*R* form	Basic/effective	Definitions
Lawrence *et al*. [43]	Population	IID [57]	Deterministic	*R* = *βN*/*γ*	Basic	*β* transmission rate
						*N* population size
						*γ* recovery rate

O'Neill and Marks [20]	School	Absence information and questionnaires	Discrete time Reed-Frost			

Vanderpas *et al*. [48]	Long- term care facility	Incidence data from stool samples	Deterministic	*R* = *T*_g_(*y*(*t*)/*t*) + 1	Effective	*T*_g_ Generation time *y*(*t*) incidence at time *t*

Heijne *et al*. [47]	Scout jamboree	Case numbers and onset times	N/A	*R*(*t*) = (*G*(*t*_h_ − *t*) + (1 − *G*(*t*_h_ − *t*))*ρ*)*R*_u_	Effective	*t*_h_ time enhanced hygiene measure are introduced *G* CDF of generation time distribution *ρ* relative reduction in *R* due to hygiene measures *R*_u_ effective reproduction number without hygiene measures

Sukhrie *et al*. [52]	Hospital and care homes	Surveys and incidence data from stool samples	Serial intervals and transmission trees	N/A	Effective	

Simmons *et al*. [35]	Population	IID2 [57]	Deterministic	*R* = *ρ*(−*TE*^−1^)	Basic	lead eigenvalue of matrix *M*
						*T* transmission matrix
						*E* transition matrix.

Lopman *et al*. [10]	Population	IID2 [57]	Deterministic			

Milbrath *et al*. [31]	Population	Published shedding durations from challenge studies and outbreak investigations	Deterministic and stochastic	*R* = *βN*((1 − *ρ*/*γ*_R_) + (*ρ*/*γ*_L_))	Basic	*β* transmission rate

						*γ*_*i*_ recovery of long, *i* = *L*, or regular, *i* = *R*, shedders
						*ρ* proportion of population that are long shedders
Zelner *et al*. [46]	Households	Incidence data from phone surveys	Discrete stochastic			

Lane [59]	Population	IID2 [57]	Deterministic			

O'Dea *et al*. [53]	Multiple hospitals	Survey and simulated data	Stochastic	*R* = (*r*_patient_ + *r*_staff_)/*μ*_patient_	Basic	*r*_type_ initial growth of type-infected population
						*μ*_patient_ recovery rate of patient-infected population

Bartsch *et al*. [54]	Multiple hospitals	Simulated data	Agent-based Reed-Frost	Estimates used from Simmons *et al*. and Vanderpas *et al*.		

Steele *et al*. [69]	Population	Hospitalisation counts	Deterministic	*R* = *ρ*(−*TE*^−1^)	Basic	*ρ*(*M*) lead eigenvalue of matrix *M*
						*T* transmission matrix
						*E* transition matrix

Matsuyama *et al*. [21]	Population	Outbreak case notifications	Deterministic	*R*_y_ = 1/1 − *q*	Effective	*q* proportion of human-to-human transmission events between outbreaks
						*R*_y_ yearly effective reproduction number

## IN OUTBREAK SETTINGS

Outbreaks are typically defined as a number of cases that is in excess of what would be commonly seen in that season or location. Norovirus is commonly associated with outbreaks in healthcare settings such as hospitals or long-term care facilities (LTCFs). However, outbreaks are also regularly documented on cruise ships, planes, schools and restaurants [20, 24, 25, 46, 47]. Models of norovirus in outbreak settings usually utilise primary data which may be extremely heterogeneous. This is because outbreaks affect a subset of the population that may not be representative of the whole; for example, an outbreak in a school will only affect certain age groups. As such, the model dynamics and value of any estimated reproduction number will be highly specific to the particular dataset and epidemiological setting. The nature of the data and population in these settings mean that stochastic effects are extremely important. In a small population such as a hospital ward, the influence of a random effect could spell the end of the outbreak. As such, models of outbreaks are generally stochastic which means that transitions are defined by probability distributions rather than fixed values, allowing for different dynamics at every simulation. An exception to this generalisation is the deterministic model of Vanderpas *et al*., which was estimated using stool confirmed case numbers from an outbreak in four wards of a LTCF [48]. An issue with a deterministic model for such a small population is that there is a high probability of stochastic epidemic extinction which will not be accounted for. As such, the predicted case numbers of Vanderpas *et al*. may be overestimated [48]. No information on susceptibility was available, or informed this estimate, thus it should be considered as an effective reproduction number. We would therefore expect that the corresponding value of the basic reproduction ratio, accounting for pre-existing immunity, would be greater for this outbreak.

Norovirus is colloquially known as winter vomiting disease and vomiting has been shown to be important when modelling outbreaks. A vomiting incident may not be noticeable in a large population, but in a small, closed population, vomiting can drastically affect the course of an epidemic. Vomiting incidents can produce airborne viral particles and contaminate *fomites*, surfaces that can act as vehicles of transmission [49, 50]. Models explicitly including vomiting, or an altered infectiousness profile, have been found to more accurately represent outbreak data. In a school, a model including infections caused explicitly by vomiting incidents better reflected survey data on absences and self-reported onset times [20]. Similarly, in a household setting, an initial spike in infectiousness of infected individuals was shown to be a key aspect to reflect survey data [46]. As such, the inclusion of heterogeneity in symptomatic individuals is an important consideration when estimating models from outbreak data. However, it should be noted that a varying infectiousness profile was found to be less influential in models estimated from data at the community level [31].

In healthcare settings, outbreaks of norovirus are particularly prevalent. This may be partly due to the immunocompromised status of the patient population or a high number of individuals who may struggle to sustain hygiene levels due to their condition. Additionally, it may be due to the increased rate of hospitalisation due to norovirus gastroenteritis experienced by individuals with pre-existing chronic medical conditions [51]. However, we might also expect the level of hygiene in these settings to be above average, at least for healthcare workers. This leads to the question of whether healthcare workers contribute to transmission in the same way as patients do. Through comparison of effective reproduction numbers for individuals in nosocomial outbreaks, Sukhrie *et al*. found that transmission was driven by patients rather than healthcare workers, particularly when patients were symptomatic [52]. The values were calculated from estimated onset times and serial intervals using a Bayesian framework for symptomatic or asymptomatic patients or healthcare workers. The highest effective reproduction numbers were found for symptomatic patients and the lowest, deemed negligible, were found for asymptomatic healthcare workers. Therefore, whilst the specific inclusion of asymptomatic individuals in models of norovirus may be an important consideration in the community in general, discussed later, the differentiation may not be required for models of nosocomial transmission as their specific contribution is very small.

An issue with outbreaks in healthcare settings or outbreaks in general are the size of datasets and specificity of estimated parameters to the specific location. Multiple outbreak data may be used to address this problem and arrive at an estimate that applies to general healthcare settings whilst accounting for uncertainty caused by differing situations. A technique for utilising multiple outbreak data was applied by O'Dea *et al*. and shown to be improved by the inclusion of an increasing number of outbreak datasets [53]. In this situation, individual outbreaks are assumed to be separate realisations within a given setting, allowing the reproduction number to be estimated for each facility, with ranges shown in Figure 2.

However, there may be biases in reporting with larger outbreaks reported over smaller ones. One such outbreak on a larger scale was that in a scout jamboree, Heijne *et al*. examined transmission of norovirus in an outbreak where 329 individuals became infected. Heijne *et al*. used this dataset to estimate reproduction numbers from generation times and to establish the effectiveness of hygiene measures. The range of values extended from a minimum of just over 2 to a maximum of over 14 without health interventions. The three values plotted in Figure 2 show the estimate with hygiene interventions of 2·13, the estimate at the start of the epidemic of 7·26 and the estimate of the effective reproduction number in the absence of any intervention of 14·05. The high values could be in part due to the generally younger population of individuals at the jamboree both because of their immunological naïvety and their levels of hygiene.

Whilst outbreaks in hospitals and LTCFs appear to be isolated occurrences, healthcare facilities form a vibrant network. Bartsch *et al*. compared transmission settings through values of the basic reproduction number taken from Simmons *et al*. and Vanderpas *et al*. to assess the potential for norovirus to jump between nodes in a hospital network via patient sharing [35, 48, 54]. They found this transferral of patients propagates epidemics between facilities with a probability directly related to the basic reproduction number [54]. Therefore, the reproduction number is not only an assessment of risk within a facility but also the risk of between facility spread.

## IN THE COMMUNITY

Community- or population-level models apply on a large scale, for example, in a city or country. In general, models at community level are more likely to be deterministic and this is true for norovirus models; only Milbrath *et al*. consider the effect of stochasticity [31]. This pragmatic choice trades off the computational and analytical benefits of deterministic models against more realistic, but complex, stochastic formulations [55]. However, the validity of this argument depends on the nature of the stochasticity driving the system. Whilst the impact of demographic stochacity – uncertainty in the timing of events occurring at a constant rate – is generally less in large populations, so-called environmental sources of stochasticity may still dominate dynamics in a large population and more subtle effects like stochastic resonance may further undermine the common assumption of equivalence between deterministic and stochastic models [56]. The referenced works all use the SEIR structure with an additional compartment for asymptomatic individuals. Another similarity is that most of the shown works are estimated from the second Infectious Intestinal Disease, IID2, study data from the UK [57]; Lawrence *et al*. use data from the first IID study.

The infectious intestinal disease surveys examine reports of infectious intestinal disease in the community as reported by recruited individuals, general practitioners (GPs), practise nurses, through database searches and through retrospective telephone surveys in the UK [58]. Infectious intestinal disease is extremely under-reported and this is partly due to a reduction in individuals visiting their GPs over time; between the first and second IID surveys, the number of GP visits for gastroenteritis fell by 50%. Yet, the rate of IID was 43% higher in 2008, at the time of the second IID survey, than at the time of first. Norovirus was the most common pathogen presenting to GPs in the survey with 2 905 278 cases in 2008–2009; however Tam *et al*. estimate that for every one case reported to national surveillance, 288 more occur in the community without being reported [57]. The IID studies give a comprehensive overview of reported cases and the number of cases that may go unaccounted for has been estimated. As such, they provide a wealth of information for model estimation that has been rightly exploited over recent years.

The first work in Figure 1, Lawrence *et al*. utilised IID data to estimate the proportional contribution of food-borne transmission to norovirus prevalence [43]. In all studies shown in Figure 1, primary transmission is assumed to be direct person-to-person. However, this emphasis fails to acknowledge the potential importance of food-borne transmission to case numbers [58] as well as the role of environmental contamination [23]. Initial efforts were aimed at defining a limit for the number of exogenously generated cases required to maintain the disease in the population [43]. This quantity was derived from the basic reproduction number calculated from the estimated model (see Table 1). The value was highly sensitive to the duration of immunity experienced by recovered individuals and the limit was found to be between 0·23 and 1·8 million cases per year in England. The wide variation shown in these two values was as a result of assumed under-reporting in the first IID survey, a problem that was addressed in complementary work by Lane using IID2 [59]. The updated infectious intestinal disease survey data led to a reassessment of the proportion of asymptomatic individuals. In the first study, the estimated proportion of asymptomatic infected individuals is 0·3%; however, Lane substantially revised this estimate to 12% based on expert opinion at the Food Standards Agency rather than estimation from the IID survey data. The new estimate not only prompted consideration of the effect of asymptomatic norovirus infection in the community but altered the estimates of food-borne contribution to transmission to 2·9 million/year in England.

The difference between human-to-human and food-borne cases were further assessed in Japan by Matsuyama *et al*. [21]. They linked final epidemic sizes with effective reproduction numbers to measure transmissability of norovirus through different transmission routes. Using outbreak data from 2000 to 2016, they evaluated the yearly effective reproduction number under different sampling assumptions. They concluded that, in Japan, person-to-person transmission of norovirus is increasing and with it, the effective reproduction number.

Asymptomatic individuals have been found to have negligible effects on transmission in certain outbreak settings; however, the models estimated from the IID surveys imply that it may be important in the community and there have been instances where asymptomatic individuals have seeded outbreaks. In all the cited works shown in Figure 1, asymptomatic individuals are explicitly included at the community level. This is partly as a result of the work of Simmons *et al*. who considered differing model formulations with varying levels of contribution from asymptomatic individuals and asymptomatic reinfection of recovered individuals [35]. The IID2 survey data are best reflected by models including both asymptomatic individuals *and* asymptomatic reinfection of recovered individuals. Furthermore, asymptomatic individuals may act as a reservoir of infection within the population for different transmission settings [10]. As such, the inclusion of an asymptomatic compartment within community models for norovirus is likely to be important.

The real focus of the different model formulations in the work of Simmons *et al*. is an assessment of the duration of immunity [35]. They found that the duration of immunity was not sensitive to asymptomatic reinfection and generally much higher than previous estimates. In the work of Lane and Lawrence *et al*., the duration of immunity was assumed to be between 6 months and a year; however, Simmons *et al*. found the duration to be in the region of 5 years. This difference could be due, in part, to differences in model structure, for example, Simmons *et al*. assume an age-structured model in all formulations. It may also be related to additional use of challenge study data to estimate the proportion of the population that is immune to infection. This more varied approach to estimation leads to a more robust result which has been used in many of the subsequent studies. It should also be noted that through the construction and estimation of different norovirus transmission models, different values of *R*_0_ were calculated with values ranging from 1·64 to over 7. These were heavily influenced by the model structure and particularly the weighting of asymptomatic and latent individual infectiousness.

The weighting of contribution from asymptomatic and exposed individuals may also be important when modelling norovirus transmission. Viral particles shed from an individual may be viable for infection [60]. Additionally, it has been found that asymptomatic individuals shed a 1–2 log smaller concentration of viral particles in their stool than symptomatic individuals but may shed for a long duration [3, 61]. Therefore, it is important to establish the relative shedding quantities of individuals in different states in mathematical models. Initially, the contribution of asymptomatic and symptomatic individuals was considered equal in the work of Lawrence *et al*.; however, the asymptomatic carriage was assumed to be so low that the model would have not appeared sensitive to any scaling of asymptomatic contribution. When Lane reassessed the asymptomatic carriage in the population, he also discussed different weights of contribution from asymptomatic individuals but assumed equal contribution. A fuller examination of the difference in weighting for asymptomatic and latent individuals was conducted in the work of Simmons *et al*.. In this case, a model where asymptomatic and exposed individuals are 5% as infectious as symptomatic individuals was a better reflection of the IID2 data than one with either a 25% or 0% weighting. They did not assess the effect of a 100% weighting; however, the basic reproduction number estimate was higher when asymptomatic individuals had a greater contribution to the force of infection, a trend seen also for rotavirus [62]. As such, the contribution of asymptomatic individuals to the basic reproduction number is important and yet hugely uncertain as they are a large and unobserved component of the system.

There are operational differences between the shedding of asymptomatic and symptomatic individuals. As such, the explicit inclusion of variation in shedding duration was found to be extremely important. Long shedding individuals, either defined functionally or operationally, can increase the value of *R*_0_ by 50–80%, see Figure 2 for values [31]. Additionally, individuals shedding for a longer duration may increase the probability of an epidemic. As such, the inclusion of asymptomatic-but-shedding individuals with heterogeneous shedding durations may be necessary to be consistent with the data from outbreak investigations and challenge studies. It should be noted that Milbrath *et al*. use challenge studies and outbreak investigations to estimate their community model which may lead to overestimation of shedding duration. However, it has been found that robust estimation of models using viral shedding data can be comparable with community-level estimates [63].

## DISCUSSION

By examining published mathematical models of norovirus transmission, we found a divided picture of current modelling approaches, dictated by setting and context. At a population level, the deterministic model structures were largely similar across different settings, and most were underpinned by data from the IID survey [64]. In outbreak settings, the models are far more diverse, reflecting the setting and population they are approximating, although these models generally (and appropriately) took a stochastic approach. With these disparities in model approach and radical differences in the outbreak contexts, there is likely to be some variation in reproduction number estimates. However, we found that the values of the basic reproduction number for norovirus varied widely, from 1·1 to 7·2. In general, population-based estimates of *R*_0_ are around 2, with higher values estimated for particular outbreaks. Whilst norovirus in a subset of a population in a confined environment may have a different transmission potential to the population as a whole, other data sources and characteristics of norovirus suggest that the basic reproduction number may be underestimated at the population level. Examination of population seroprevalence data shows that the first infection with norovirus occurs very early in life, before 5 years of age. As such, a simple calculation, given the mean age at first infection is 2 years and assuming the duration of immunity is 5 years, see Simmons *et al*., gives *R*_0_ ≈ (*A* + *D*/*A*) = 3·5 [42]. Additionally, the low infectious dose, potentially long shedding durations and the high level of asymptomatic carriage imply that transmission potential is extremely high. The best fit estimate of *R*_0_ for rotavirus, an enteric pathogen with a similarly low infectious dose, for comparison is 26·2 in [62], with estimates ranging from 1·23 to 26·2, but between 23·3 and 191 in [65].

Further research could help to address such important inconsistencies and information gaps identified in this review. In particular, some features of the natural history of norovirus infection are not well described. A more thorough examination of seroprevalence data in different settings is warranted and may well improve our estimates of *R*_0_ [66]. The models that we have reviewed have also made some key assumptions regarding asymptomatic individuals. The proportion of individuals without symptoms has been assumed to be as low as 0·3% and as high as 30% [10, 35, 43, 52, 59]. This is an important distinction as asymptomatic individuals have been found to shed a 1–2 log smaller concentration of viral particles and can shed for long durations [3, 31, 61]. Empirical evidence on the role of asymptomatic individuals in ongoing transmission will better inform future models. The duration of immunity to norovirus is also not well established for norovirus. In the earliest studies, immunity was assumed to be complete and short-lived, in the region of 6 months to a year leading to an approximate value or *R*_0_ of 1·25 by the same, simple calculation. Yet, in later studies, immunity was robustly shown to be leaky and last far longer, around 5 years. The difference is significant as a longer immune period will lead to a force of infection that is proportionally higher in order for the system to see the same numbers of reported cases; as such, the duration of immunity can have a direct effect on the basic reproduction number. Another remedy for inconsistencies may lie in model comparison similar to that of Pitzer *et al*., where different approaches were brought together for the same dataset [62]. A final, and vital, omission from the current literature are models and estimates applying to lower and middle-income countries (LMIC). There have been some studies into the differences in norovirus across varying country profiles, for example [19]; however, there have been no mathematical models of the transmission of norovirus in LMIC settings. We may expect the estimates of key quantities such as the basic reproduction number to vary substantially; we know that there are substantial differences in contact structures and environmental hygiene. The incidence of norovirus gastroenteritis seems universally high in children in developed and developing countries, with an incidence or around 20% per year [67, 68]. However, there is still reason to think that the *R*_0_ is higher in developing country settings because of higher exposure through (a) person-to-person transmission because of greater population density/contact rates or (b) environmental/food transmission because of lower levels of clean water, sanitation and hygiene.

Establishing more robust models for norovirus is particularly important in the context of the development of new vaccines. Questions regarding the potential impact of vaccination and their optimal use can be efficiently addressed using mathematical modelling. Mathematical models are vital for examining these questions because a much broader range of strategies (and model assumptions) can be tested in silico than in real-life clinical trials. The basic reproduction number can be central to this as it captures the efforts required to control an infection. An initial modelling study, conducted by Steele *et al*., suggested that targeting infants may be the most efficacious strategy [69]. However, updated estimation and examination of more varied control strategies may give us further insight into regulating norovirus. For example, the strong seasonality exhibited by norovirus would affect time-dependant control measures and, whilst seasonality has been included in models, the interplay between it and intervention measures have not yet been examined. An examination of the effect of temporal control measures on seasonal norovirus could find interesting dynamics such as changing periodicity or varying synchrony either spatially or temporally. Given the varying setting and approaches, there are currently a myriad of model formulations for norovirus. However, we have highlighted some key knowledge gaps that, if filled, could give a far better outlook for modelling and therefore understanding norovirus transmission.

## Supplementary material

For supplementary material accompanying this paper visit https://doi.org/10.1017/S0950268817002692. click here to view supplementary material
